# Association of rs7216389 Polymorphism in Gasdermin B (GSDMB) With Childhood Asthma: A Case-Control Study

**DOI:** 10.7759/cureus.76937

**Published:** 2025-01-05

**Authors:** Qudsia U Khan, Afreen Bano, Ismail Mazhar, Aimen B Asif, Muhammad Ibrahim Tahir, Amaan Ahmad, Arhamah Zahid, Maryam Ahmed Khan

**Affiliations:** 1 Department of Physiology, CMH Lahore Medical College and Institute of Dentistry, Lahore, PAK; 2 Department of Microbiology and Parasitology, Lincoln University College, Petaling Jaya, MYS; 3 Department of Medicine, CMH Lahore Medical College and Institute of Dentistry, Lahore, PAK; 4 Department of Medicine, Lahore Medical and Dental College, Lahore, PAK; 5 Department of Anatomy, Fazaia Medical College, Islamabad, PAK; 6 Department of Medicine, Fazaia Medical College, Islamabad, PAK

**Keywords:** asthma pathogenesis, childhood asthma, gasdermin b, genetic polymorphism, gsdmb, rs7216389 variant

## Abstract

Objective

This study examines the association between the gasdermin B (GSDMB) gene variant rs7216389 and childhood asthma, with a focus on gender-based differences, environmental factors, and lung function measurements in affected children. It highlights the growing prevalence of childhood asthma, its unique features compared to adult-onset asthma, and the substantial healthcare burden it imposes, especially during exacerbations.

Methods

A case-control study was conducted over 18 months at CMH Lahore, UHS, and Children’s Hospital, including 200 participants (100 asthmatics, 100 controls) aged three to 18. Blood samples were analyzed for genetic factors. IBM SPSS Statistics for Windows, Version 25.0 (Released 2017; IBM Corp., Armonk, NY, USA) was used for statistical analysis, with significance at p < 0.05. Ethical approval and informed consent were obtained.

Results

The study identifies the GSDMB variant rs7216389 as a potential genetic marker for asthma, underscoring its association with the severity of the condition in children. It highlights the challenges of translating genetic findings into clinical practice while emphasizing the therapeutic potential of targeting these genetic markers. The study also sheds light on healthcare costs and the distinctive clinical features of pediatric asthma, further contextualizing its impact.

Conclusions

This article provides a comprehensive overview of asthma pathogenesis, emphasizing the significance of genetic markers like rs7216389 in the GSDMB gene. It advocates for further research to unravel the complex interplay of genetic, environmental, and immune factors in childhood asthma, intending to develop targeted therapeutic interventions.

## Introduction

Asthma is the most common chronic condition in children and one of the most common disorders that affect them [1]. Recent data from the World Health Organization indicates that the prevalence of asthma is increasing worldwide, with epidemiological studies estimating that respiratory allergies affect 15-30% of the global population, and asthma impacts 3.5-20% [2]. In Pakistan, childhood asthma is a significant public health issue, with prevalence rates varying between 4% and 31.5% across different cities [3]. According to research, acute treatment, ER visits, and hospital stays account for 87% of asthma-related expenses [4].

Exacerbations are defined by the Global Initiative for Asthma (GINA) as times when a patient’s condition worsens, and a change in care is necessary [4]. The symptoms that worsen during these episodes are shortness of breath, coughing, wheezing, chest tightness, and decreased forced expiratory volume in 1 second (FEV1)/forced vital capacity (FVC) ratio [5]. Even after much research, asthma is still one of the most difficult conditions to treat globally, with numerous unresolved issues about its molecular causes and available therapies [6]. The features and consequences of asthma differ according to age and gender. Asthma in children is frequently atopic and is linked to increased bronchial reactivity, decreased FEV1/FVC ratio, raised IgE levels, and allergen exposure [6]. Women are more likely than men to have adult-onset asthma, which is less commonly linked to allergens [7].

In the case of childhood asthma, common causative factors include genetic predisposition, exposure to indoor allergens (such as dust mites, pet dander, and mold), outdoor air pollution, tobacco smoke exposure, respiratory infections, and a history of atopy or allergic conditions [8]. Additionally, tobacco use, indoor and outdoor air pollution exposure, and occupational irritants are major risk factors for adult-onset asthma [9]. While chronic obstructive pulmonary disease (COPD) affects 251 million individuals worldwide, asthma affects 339 million people and is responsible for over 1,000 fatalities per day [10]. By 2030, it is estimated that COPD and related illnesses will account for 4.5 million deaths annually worldwide, with 90% of COPD-related deaths occurring in low- and middle-income nations [2]. Asthma affects approximately 7.5 million adults and 15 million children in Pakistan, accounting for 2.1% and 4.3% of the population, respectively [4,11]. One-fourth of patients in primary healthcare facilities have one of the two serious respiratory disorders, COPD or asthma [11].

More than 150 genetic markers associated with asthma have been found by genome-wide association studies (GWAS), providing insight into the underlying causes of the condition in adults and children [12]. Research has looked into the connection between childhood asthma and the ORMDL3 gene’s rs7216389 polymorphism, which is found at 17q21. The rs7216389 polymorphism influences asthma in children by altering the expression of genes like ORMDL3 and gasdermin B (GSDMB), which are involved in immune regulation and airway inflammation. The T allele is associated with increased gene expression, leading to higher asthma risk. Moreover, this single-nucleotide polymorphism (SNP) interacts with environmental factors such as allergens and smoke, further contributing to asthma development in children [13,14].

T-cells and the airway epithelium primarily cause childhood-onset asthma, and research indicates that the gene and tissue specificity of asthma in children and adults varies [15]. The chromosomal region 17q12-q21, which was first discovered four years ago and is a 6-mb region linked to childhood asthma, has the greatest evidence [16]. Similar to GWAS results, research on the genetic association between 17q12-q21 and asthma mostly focuses on European populations. However, Asian ethnicities also have a sizeable heritage [17]. 17q12-q21 SNPs are consistently linked to asthma in cross-population studies involving African Americans, Asian Americans, and multiethnic groups [18,19]. It is difficult to identify the precise changes and genes causing elevated asthma risk because of the multitude of connections these two regions exhibit with pediatric asthma [5].

The GSDMB gene is involved in the regulation of immune responses, particularly in the activation of inflammasomes, which play a crucial role in airway inflammation seen in asthma. Variations in the rs7216389 polymorphism of GSDMB can influence its expression, potentially altering immune cell function and contributing to asthma pathogenesis. Studies have shown that higher expression levels of GSDMB correlate with increased asthma severity and a greater number of exacerbations [20]. Additionally, GSDMB has been identified as a key genetic determinant potentially influenced by a dosage effect, meaning that the number of risk alleles can affect the severity of asthma. This suggests that individuals with multiple copies of the risk allele may have a higher risk of developing asthma or experiencing more severe symptoms [21].

The goal is to identify the function of the GSDMB gene in inflammatory and immunological processes and investigate how it affects asthma differently in adults and children. The article also aims to investigate gene regulatory mechanisms, the biological impact of rs7216389 on the development of pediatric asthma, and the prevalence of GSDMB in different cell types. By providing insights that may guide future research and treatment approaches, this comprehensive analysis seeks to elucidate how GSDMB genetic variants impact asthma susceptibility.

## Materials and methods

Following clinical practice guidelines and adhering to the Declaration of Helsinki, a case-control research study was conducted at CMH Lahore Medical and Dental College and Children’s Hospital Lahore. The trial ran for 18 months, from March 3, 2023 to May 21, 2024, after obtaining Institutional Review Board (IRB) approval on February 14, 2023 (Case #.103/ERC/CMH/LMC) from the Office of Research, Innovation, and Commercialization (ORIC), CMH Lahore Medical College and Institute of Dentistry. The sample size was calculated using the finite population correlation formula, with a 95% CI and 5% absolute precision, resulting in 200 male and female volunteers divided equally into 100 asthmatics (case group) and 100 non-asthmatics (control group). A purposive sampling method was utilized.

Participants were selected based on the GINA 2019 standards, including males and females aged three to 18 years with either a current or prior diagnosis of asthma. Sampling was performed after obtaining informed consent from participants or their guardians and necessary permissions from the administration. DNA extraction was carried out using standard protocols, followed by confirmation through DNA sequencing. The amplification refractory mutation system PCR was employed for DNA amplification, and Sanger’s method was used for sequencing to ensure high accuracy in analysis. Both PCR and DNA sequencing were conducted at the University of Health Sciences. To assess lung function, a portable clinical spirometer was used to measure spirometric parameters such as FEV1 and FVC. Additionally, Wright’s peak flow meter was employed to measure peak expiratory flow rate (PEFR), ensuring precision and ease of use across diverse settings.

The inclusion criteria for asthmatics comprised symptoms such as wheezing, coughing, and dyspnea; risk factors such as a family history of asthma or symptoms triggered by physical exercise; and radiographic evidence ruling out other lung conditions. Non-asthmatic controls, aged three to 18 years, were included if they had no history of coughing, wheezing, dyspnea, or allergies, nor any prior diagnosis of asthma. A history of pet exposure, including birds, cats, and dogs, was an additional inclusion criterion for all participants.

The exclusion criteria for asthmatics included acute lung infections, such as pneumonia, or congenital disorders, such as chronic lung disease, cystic fibrosis, congenital lobar emphysema, or COPD. Non-asthmatic controls were excluded if they exhibited any symptoms resembling asthma. Informed consent was obtained from the guardians of children with asthma before data collection. Blood samples were collected from each participant using 3 cc syringes, extracting 2 ml of blood. The samples were placed in vacutainer tubes containing EDTA, an anticoagulant, and maintained at 4°C. The collected samples were transported to the Department of Human Genetics and Molecular Biology at the University of Health Sciences, Lahore, for further analysis.

Statistical analysis

Data analysis was conducted using IBM SPSS Statistics for Windows, Version 25.0 (Released 2017; IBM Corp., Armonk, NY, USA). Quantitative variables are presented as means and SDs. Qualitative variables are reported in percentages. A p-value of <0.05 was considered statistically significant. Frequencies and percentages were calculated for categorical variables, while means and SDs were reported for quantitative variables. The association between categorical variables was evaluated using the chi-square test.

## Results

Gender distribution

**Figure 1:**
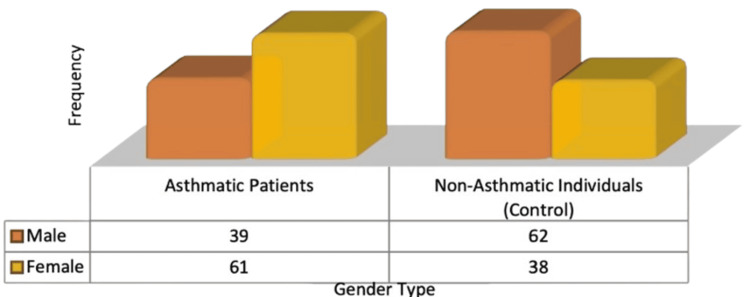
Gender-wise distribution among both groups

Figure 1 distinguishes between the gender distribution of asthmatic and non-asthmatic populations.

Comparison of asthma and non-asthma SNP profiles

The results of the chi-square test (Table 1) prove a significant relationship between asthma status and SNP variants. The chi-square test is highly significant (p < 0.001), which means that SNP variant frequency (C/C, T/C, T/T) varies significantly in asthmatic and non-asthmatic individuals.

**Table 1:** Comparison of asthma and non-asthma SNP profiles SNP, single-nucleotide polymorphism

Chi-square test			
	Value	Df	Asymptotic significance (two sided)
Pearson chi-square	200.000	3	0.000
Likelihood ratio	277.259	3	0.000
Linear-by-linear association	81.557	1	0.000
Number of valid cases	200		

SNP genotyping (rs7216389)

**Figure 2:**
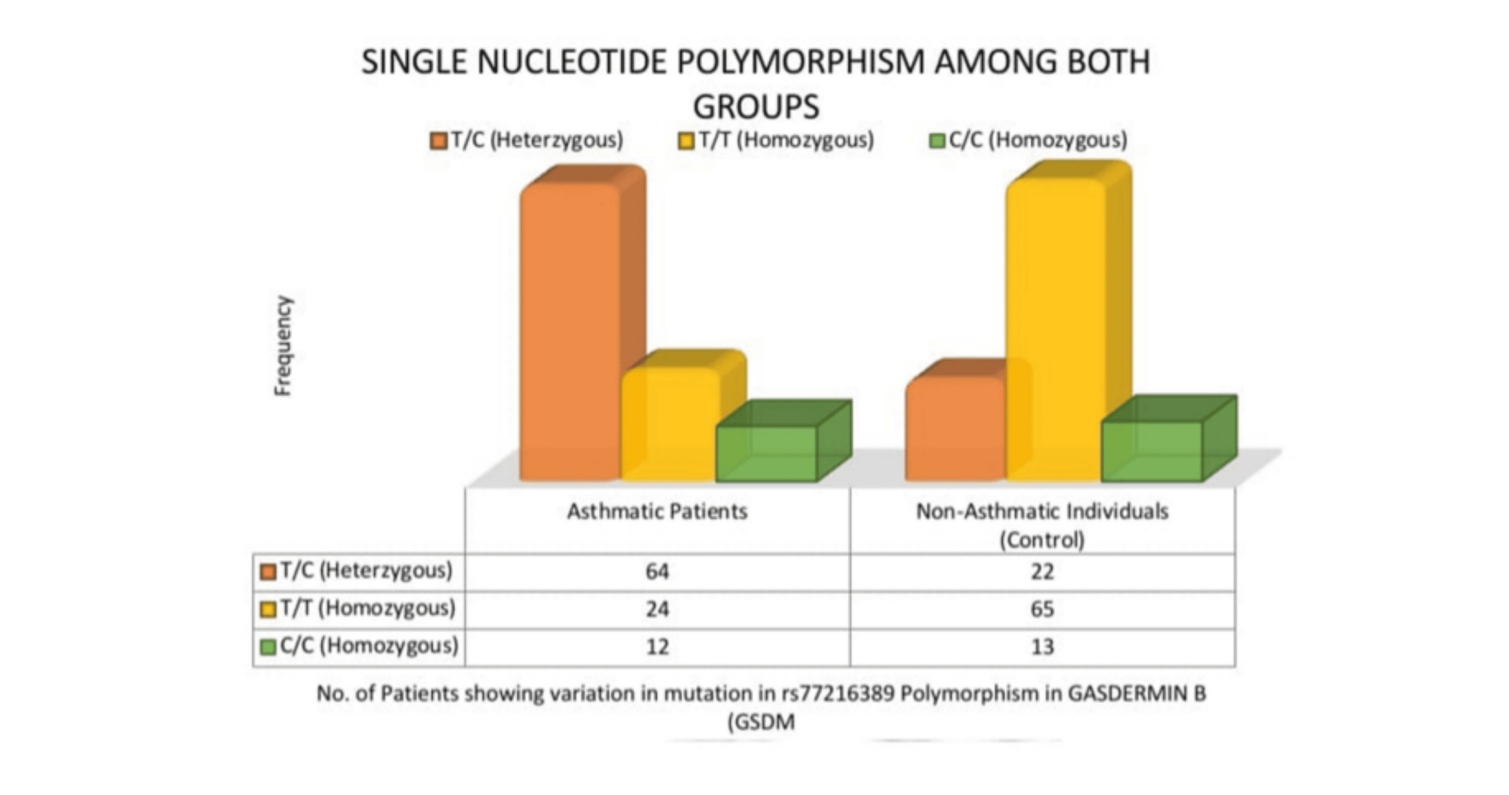
SNP among both groups SNP, single-nucleotide polymorphism

Figure 2 shows SNP genotyping results among asthmatic patients and controls.

Impact of pet exposure on asthma vs. non-asthma cases

The results from the chi-square tests in Table 2 indicate an association between the level of asthma and exposure to pets. The chi-square tests revealed a significant (p < 0.001) relationship between the variables. However, the linear-by-linear association test yielded a value of 2.970 with a p-value of 0.085, indicating that the linear relationship between exposure frequency and asthma status is nonsignificant. This suggests that while there is a general association, the specific trend of increasing or decreasing asthma severity with varying levels of exposure frequency was not statistically significant.

**Table 2:** Impact of pet exposure on asthma vs. non-asthma cases

Chi-square test			
	Value	Df	Asymptotic significance (two sided)
Pearson chi-square	62.216	3	0.000
Likelihood ratio	86.167	3	0.000
Linear-by-linear association	2.970	1	0.085
Number of valid cases	200		

Comparison of spirometry results (FEV1) in asthmatic vs. non-asthmatic groups

The chi-square tests in Table 3 establish a highly significant association between asthma status and spirometry results: FEV1. Pearson chi-square (p < 0.001) points out a significant correlation between asthma status and FEV1 outcomes. The value of the linear-by-linear association is 145.237 with a p-value < 0.001, suggesting a significant linear relationship, indicating a direct proportionality between changes in FEV1 scores and asthma status.

**Table 3:** Comparison of spirometry results (FEV1) in asthmatic vs. non-asthmatic groups FEV1, forced expiratory volume in 1 second

Chi-square test			
	Value	Df	Asymptotic significance (two sided)
Pearson chi-square	200.000	4	0.000
Likelihood ratio	277.259	4	0.000
Linear-by-linear association	145.237	1	0.000
Number of valid cases	200		

Analysis of PEFR in asthma vs. non-asthma groups

The chi-square tests in Table 4 show a relationship between the variables, such as asthma status, physical evaluation and exercise capacity, and PEFR. Pearson chi-square (p < 0.001) and the likelihood ratio (p < 0.001) both show the presence of a connection between asthma status and PEFR measures. The results strongly substantiate the proposition that PEFR is positively linked to asthma; this captures how asthma status affects lung function/PEFR and expiratory flow.

**Table 4:** Analysis of PEFR in asthma vs. non-asthma groups PEFR, peak expiratory flow rate

Chi-square test			
	Value	Df	Asymptotic significance (two sided)
Pearson chi-square	113.609	2	0.000
Likelihood ratio	129.325	2	0.000
Linear-by-linear association	98.483	1	0.000
Number of valid cases	200		

Association of gender in asthmatic vs. non-asthmatic groups

Table 5 also shows there is a relationship between asthma status and gender. Pearson chi-square (p = 0.005) and the likelihood ratio (p < 0.005) show that gender has a significant association with asthma status. The linear-by-linear association (p = 0.005) shows the presence of a significant linear relationship, which gives evidence and supports the argument that gender differences are meaningful in explaining asthma status. The association is also corroborated by Fisher’s exact test, with the respective p-values being 0.007 (two sided) and 0.004 (p = 0.001) one tailed, analyzing the result in a two-by-two contingency table, which shows the strength of the result.

**Table 5:** Association of gender in asthmatic vs. non-asthmatic groups

Chi-square test					
	Value	Df	Asymptotic significance (two sided)	Exact sig. (two sided)	Exact sig. (one sided)
Pearson chi-square	8.013	1	0.005		
Continuity correction	7.232	1	0.007		
Likelihood ratio	8.068	1	0.005		
Fisher’s exact test				0.007	0.004
Linear-by-linear association	7.973	1	0.005		
Number of valid cases	200				

## Discussion

This study examined whether children with asthma have the polymorphism rs7216389 in the GSDMB gene. Research on the genetic and environmental factors that may influence childhood asthma, which affects millions of people worldwide and whose susceptibility is influenced by a delicate balance between nature and nurture, is complemented by this work [20,21]. Sixty-one percent of the participants with asthma were female. However, it was shown that 62% of the non-asthmatic control group was male. Table 5 demonstrates that there is a strong correlation between gender and asthma status, indicating that women were more likely to have asthma. Studies have shown that women are more likely than men to develop asthma, with differences in gene expression, epigenetic changes, and environmental reactions contributing to this disparity [22]. For instance, research indicates that adult women have an increased prevalence and severity of asthma compared to men. Additionally, epigenetic modifications have been associated with asthma, suggesting that environmental factors may influence gene expression [23].

This study demonstrates that children with asthma have a significantly lower mean FEV1 and a significantly larger percentage of patients in the yellow and red zones of PEFR by comparing the differences in lung function between children with and without asthma. Previous studies have shown that inflammation of the airways and BHR frequently impair pulmonary function in children with asthma [24,25]. FEV1 has been determined to be a typical measure of asthma severity and to have a substantial positive correlation with both the frequency of exacerbations and clinical symptoms. Additionally, this study supports the notion that FEV1 has a high test-retest reliability for assessing asthma morbidity [26]. With 67% of the current asthmatic patient sample having FEV1 < PFEV1 compared to 4% in the normal control group, the current investigation supports the previously noted higher prevalence of decreased pulmonary function in the asthmatic community. Numerous studies emphasize the importance of spirometry as a crucial diagnostic and monitoring tool for asthma, particularly in children. For instance, spirometry is commonly indicated for children with chronic cough, and persistent wheezing, and the diagnosis and monitoring of asthma [27]. Additionally, spirometry plays an important role in the assessment and long-term monitoring of patients with asthma, aiding in clinical improvement and changes in spirometry measurements with treatment in children [28]. These findings underscore the clinical value of spirometry in differentiating between children with and without asthma in both research and real-world contexts [29].

Environmental factors, such as exposure to pets, significantly influenced the prevalence of asthma in this group. For example, a study found that early-life cat and dog ownership ranged from 12% to 45% and 7% to 47%, respectively, with asthma prevalence ranging from 2% to 20%. However, no overall association was found between either cat or dog ownership and asthma [30]. Another study indicated that keeping a cat in the first year of life increases the risk of sensitization to cat allergens, but keeping a cat or a dog after the first year of life was not associated with sensitization to those allergens [31]. These findings suggest that while pet exposure may influence asthma prevalence, the timing and degree of exposure are critical factors in determining the risk. Pet ownership has been classified as both protective and risk factors for asthma, depending on characteristics including genetic background and exposure age [27]. The exposure rates shown here, which showed that children with asthma had higher rates of occasional, not frequent, pet exposure, may indicate that sporadic exposure may make a child more sensitive rather than desensitized or immune; this is consistent with similar findings from other studies [28,29].

The functional gene GSDMB, located on chromosome 17q12-21, has been shown in prior research to directly contribute to the immunological response in asthma, including inflammation and epithelial cell function control. According to reports, GSDMB may control the release of TSLP, IL-33, and IL-25, which are critical for the pathophysiology of asthma, by encouraging the infiltration of neutrophils and eosinophils in the inflamed airways; this is more pronounced in individuals with the T/C or T/T genotype [30]. Studies in children also have demonstrated that polymorphism rs7216389 carriers exhibit high ORMDL3 gene expression in airway cells, predisposing them to ER stress and disturbed calcium signaling, which are connected to inflammation processes in asthma [31].

Additionally, recent studies have shown a potential gene-gene interaction between ORMDL3 and GSDMB, with co-expression of both genes linked to increased asthma susceptibility. Specifically, polymorphisms in both genes may contribute synergistically to airway inflammation and remodeling, further enhancing the risk of asthma development [32,33]. Significant genotyping evidence for the GSDMB gene polymorphism rs7216389 indicates a substantial correlation between the SNP variant and asthma condition (p < 0.001), which is corroborated by the likelihood ratio test chi-square 277.259. The statistical significance of this association suggests that this SNP is a major risk factor for childhood asthma. Nevertheless, the polymorphism has also been linked to the disease by genetic investigations conducted in other populations [34]. Specifically, a T/C heterozygous genotype was shown to be most common in patients with asthma (64%), suggesting that this genotype may be linked to increased susceptibility to the development of asthma. Studies have shown that the T/C genotype of rs7216389 may interact with specific environmental factors such as allergen exposure (e.g., dust mites and pet dander) and smoke exposure, which can exacerbate airway inflammation. For instance, individuals carrying the T/C genotype who are exposed to secondhand smoke show a heightened risk of asthma symptoms due to increased gene expression of ORMDL3, which is involved in airway remodeling and inflammation [35,36]. Additionally, early-life exposure to cats has been associated with a decreased risk of asthma in individuals carrying the rs7216389 genotype, suggesting a gene-environment interaction that modulates asthma risk [37]. These findings highlight the complex interplay between genetic predisposition and environmental exposures in the development and exacerbation of asthma.

Research has indicated that individuals with the rs7216389 polymorphism may have epigenetic modifications, such as altered methylation patterns at the GSDMB locus, which can intensify the production of inflammatory genes in reaction to allergens or pollutants [35]. With notable differences in the frequency of the rs7216389 variant between populations, ancestry also shapes the impact of GSDMB polymorphisms on asthma susceptibility. In comparison with the findings of the current study, we observed a similar trend where European and Hispanic populations were more likely to carry the T allele, correlating with a higher asthma prevalence. However, our study also revealed a notable variation within the East Asian population, where the frequency of the T allele was lower than reported in previous studies. This suggests that ethnicity may play a significant role in the distribution of the T allele and its association with asthma risk, warranting further investigation into ethnic-specific genetic factors [38]. According to recent research, the rs7216389 SNP causes GSDMB to express especially highly in dendritic cells and airway epithelial cells, and it plays a crucial role in regulating the immune response to inhaled allergens [39].

Another focus of the GSDMB rs7216389 polymorphism is its association with the early onset of asthma, which suggests that this sequence variant increases the risk of developing asthma symptoms earlier in life [25]. Additionally, the rs7216389 affects the response to the disease’s therapy, including medications used to manage inflammation in asthma, such as corticosteroids. Several studies have demonstrated that the action of rs7216389 genotype T/T in children may result in decreased sensitivity to corticosteroids, which may be explained by increased production of pro-inflammatory cytokines [25].

These kinds of studies have significant clinical implications because they create opportunities for prevention in high-risk populations. For children with a family history of asthma, for instance, testing for rs7216389 and other asthma-related SNPs might be incorporated into the routine examination [40]. From a pharmacogenomics standpoint, these results suggest that future developments in asthma therapy will be tailored while accounting for additional variables such as the genetic location rs7216389 [41]. This strategy would be particularly helpful for corticosteroids because research has indicated that genetic variables influence how well a patient responds to this treatment. By gaining technical insight into the GSDMB rs7216389 polymorphism in childhood asthma, significant strides have been made in our understanding of the genetics of asthma. Next-generation sequencing (NGS), which enables parallel investigation of genetic variations concerning complete genomes, is one such innovation. Asthma risk factors can be more precisely defined, and patients can be more precisely categorized for asthma risk thanks to NGS’s ability to identify uncommon variations that may impact asthma susceptibility in particular populations.

Limitations

The study primarily focuses on known genetic variations, such as rs7216389, but falls short of addressing rare genetic variations comprehensively. Additionally, nongenetic factors influencing asthma susceptibility are not thoroughly explored, leaving a gap in understanding the broader determinants of the condition. Although NGS is discussed, the findings are confined to currently identified genetic markers, limiting the scope of potential insights. Moreover, the applicability of these findings to diverse populations with varying genetic and environmental contexts remains uncertain. To enhance clinical relevance, further exploration is required to integrate rare genetic variations into practical applications.

## Conclusions

This study highlights the intricate interplay of genetic, demographic, and environmental factors in the development of childhood asthma. Genotyping for rs7216389 could help guide corticosteroid use by identifying patients who may require adjusted doses based on their genetic predisposition to inflammation. This could lead to more personalized and effective asthma treatment. Gender-based differences suggest an increased susceptibility among females, while variations in allergen and pet exposure highlight the complex role of environmental factors. The nonsignificant linear relationship between pet exposure frequency and asthma status underscores the nonlinear nature of environmental influences, suggesting that asthma development may involve threshold effects or other nonlinear dynamics. Reduced lung function remains a defining characteristic of asthma, reinforcing the need for early diagnosis and intervention.

These findings underscore the importance of a multifaceted approach to asthma prevention and treatment. Integrating genetic screening, environmental assessment, and personalized interventions holds promise for advancing individualized care and improving outcomes in managing childhood asthma.
